# Novel design and fabrication of a mechanically tunable multiband watch antenna for wireless applications

**DOI:** 10.1038/s41598-025-20435-6

**Published:** 2025-09-25

**Authors:** Ashraf S. Abdel Halim, Omnia Hamdy

**Affiliations:** 1 Department of Communication, Faculty of Engineering, Canadian International College (CIC), Cairo, Egypt; 2 Department of Engineering Applications of Laser, National Institute of Laser Enhanced Sciences, Cairo University, Giza, Egypt

**Keywords:** Multiband antenna, Tunable antenna, Wireless communications, Patch antenna, Electrical and electronic engineering, Energy infrastructure, Engineering

## Abstract

Novel design of a tunable watch antenna for both wideband wireless applications is presented in this paper. Arrows in the watch radiating patch are designed to control the bandwidth; also, a ground structure is designed beneath the radiating patch to allow multi-band operation. If the arrows of the watch mention a certain time, this allows a certain multiband, while if the time changes, it allows another different multiband; the antenna performs as a wideband antenna (WBA). Furthermore, we can obtain different frequency bands by assuming a mechanism to change the time of the watch. The antenna was examined at 1, 2, 3, and 4 o’clock in simulation, while the 1:45 design was fabricated. The S_11_ in the four simulated cases was examined to check the obtained frequency bands. The 1:45 fabricated antenna has a unique design and footprint of (diameter 30 x height 0.635) mm. The S_11_ for the simulated and measured antenna was examined. The obtained gain and efficiency were 70 dB and − 20 dB, respectively. The axial ratio was also examined and was less than 3 dB. When the position of either the minutes or hours arrow changes, the antenna acts as a WBA with different operating frequency bands. The mechanism of the proposed antenna designates it as a wideband antenna for GSM, CDMA, UMTS, LTE, Wi-MAX, 5G (Ultra-Fast & Low Latency) communications applications.

## Introduction

 Wireless communications and their applications have grown rapidly in recent decades. Most researches focus on developing both the hardware and software related to wireless communications. As wireless communication applications increase in human daily life, the need to find new frequency bands increases^1,2^. However, the antenna is considered one of the main parameters in any wireless communication system. Accordingly, It is very important to devote a great effort to design different types of antennas for different applications^3^. Antennas have different parameters, including radiation efficiency, bandwidth, return loss, and radiation pattern. Researchers in recent years have studied the effect of the antenna design on these parameters^4^. In recent years, most researchers have focused on achieving high data bit rates with low cost for (5G) communications^5–8^. The data bit rate for (5G) communication systems is 1000 times faster than that of (4G) communication systems^9^. Additionally, the 5G Radio Access Networks (RANs) would manage several 5G bands^8^. The ITU-R has considered different parameters to follow the increasing needs for the international mobile telephony (IMT) spectrum. The entire requirements 2020 criterion was completed by ITU-R ^10^. The proposed antenna covers different frequency bands, including the 5G frequency bands, GSM, CDMA, UMTS, LTE, Wi-MAX, 5G. The lower frequency band will guarantee better coverage for modern wireless communications. For frequencies lower than 6 GHz, 5G communications provide better data bit rates and wider coverage areas with outside-to-inside network coverage^11,12^.

A sub-6 GHz antenna designs in the literature heavily utilize printed antenna technology. Discrete wavelet transforms (DWTs) are utilized in orthogonal frequency division multiplexing (OFDM) systems to enhance spectral efficiency. So, wavelet transforms are designed to design antenna diversity schemes to enhance the efficiency of system performance^13–18^. New technologies resulted in small and highly effective antennas. Printed microstrip slot antennas are widely used to design antennas in this class^19–23^. Many applications for slot antennas are used in Wi-MAX, WLAN, Bluetooth, 4G LTE. Also, slot antennas are used in wireless 5G applications. In literature several different slot antenna design methods, including the: the transformer triple band slot antenna^19^, the cross-shaped slot coupler antenna^24^, the circular patch antenna with asymmetric open slots^25^, the octagonal slot antenna with U-shaped strips for UWB applications^26^, the monopole radiator with square slot and L-shaped strips^27^, the C-shaped coupled fed antenna with L-shaped monopole slot having orthogonal polarization^28^, a broad slot antenna with hypothetical resonances^29^, F-shaped slotted MIMO antenna with^30^, two monopole antennas with two rectangular etched slots and a T-shaped stub^31^, an elliptical patch antenna with an elliptical slot and dipole fed^32^, an antenna with a radiating element made of CSRR slots and fed by a meandered CPW^33^, and U-shaped slot antennas with wide band applications are among the antenna types^34,35^.

The improved high gain, efficiency, and small footprint of antenna designs continue to be an issue. There are a few drawbacks of slot antennas for 5G applications in the sub-6 GHz range, including the bigger slot size, narrow bandwidth, low gain, and low efficiency. So, low-profile antennas are necessary for 5G applications. As a result, the antenna thickness at 3.3 GHz should be around 1 mm ^14,36^. The design and implementation of multilayer watch antennas with wide bands for different wireless applications are presented in this paper. The feed for the watch radiating patch comes from a transmission cable. A ground structure allows the bandwidth to be optimized. The watch radiating region has arrows attached in the middle to allow for multi-band operation. A band-stop filter with a controllable resonant frequency can be additionally made by shortening and adjusting the length. The antenna design has a compact size of (30 × 0.635) mm, peak gain, and efficiency of about 70 dB and − 20 dB, respectively. The paper contributions of this study are summarized as follows:

- The (diameter 30 × 0.635 height) mm. The compact size of the suggested antenna construction.
- It operates throughout a wide frequency range, 0.1–6 GHz.
- By adjusting the time mentioned by the watch’s arrows, the frequency bands change.
- The proposed antenna has a realized peak gain of about 60 dB and is intended for GSM, CDMA, UMTS, LTE, Wi-MAX, 5G (Ultra-Fast & Low Latency) communications applications.
- The proposed antenna’s advantages of easy manufacturing and low profile ensured it as a strong contender for the design of multi-band antennas.
- By assuming a mechanism to control the arrow’s position, different multiband frequencies can be obtained, and the antenna becomes tunable.

The paper is organized as follows: As explained in "Methods", the suggested watch is built and simulated using the computer-simulated technology (CST) microwave package. "Results and discussion" contains the results and discussion of the obtained results. A comparison of the suggested antennas with the relevant works is shown in "Comparison with related work". Finaly, the conclusion is provided in "Conclusion"

## Methods

This section describes the design process in depth, covering the tuning mechanism, substrate choice, and antenna geometry. A brief overview of the CST Microwave Studio simulation setup is also provided. Key factors, such as the watch diameter, the width and length of the arrow structures, the shape of the hour markings, and the substrate material selection, were optimized through a series of iterative trials in order to construct the proposed watch antenna. These adjustments were necessary to guarantee compactness, effective radiation, and structural viability as well as to get the intended multiband performance.

### Proposed antenna structures

**Fig. 1:**
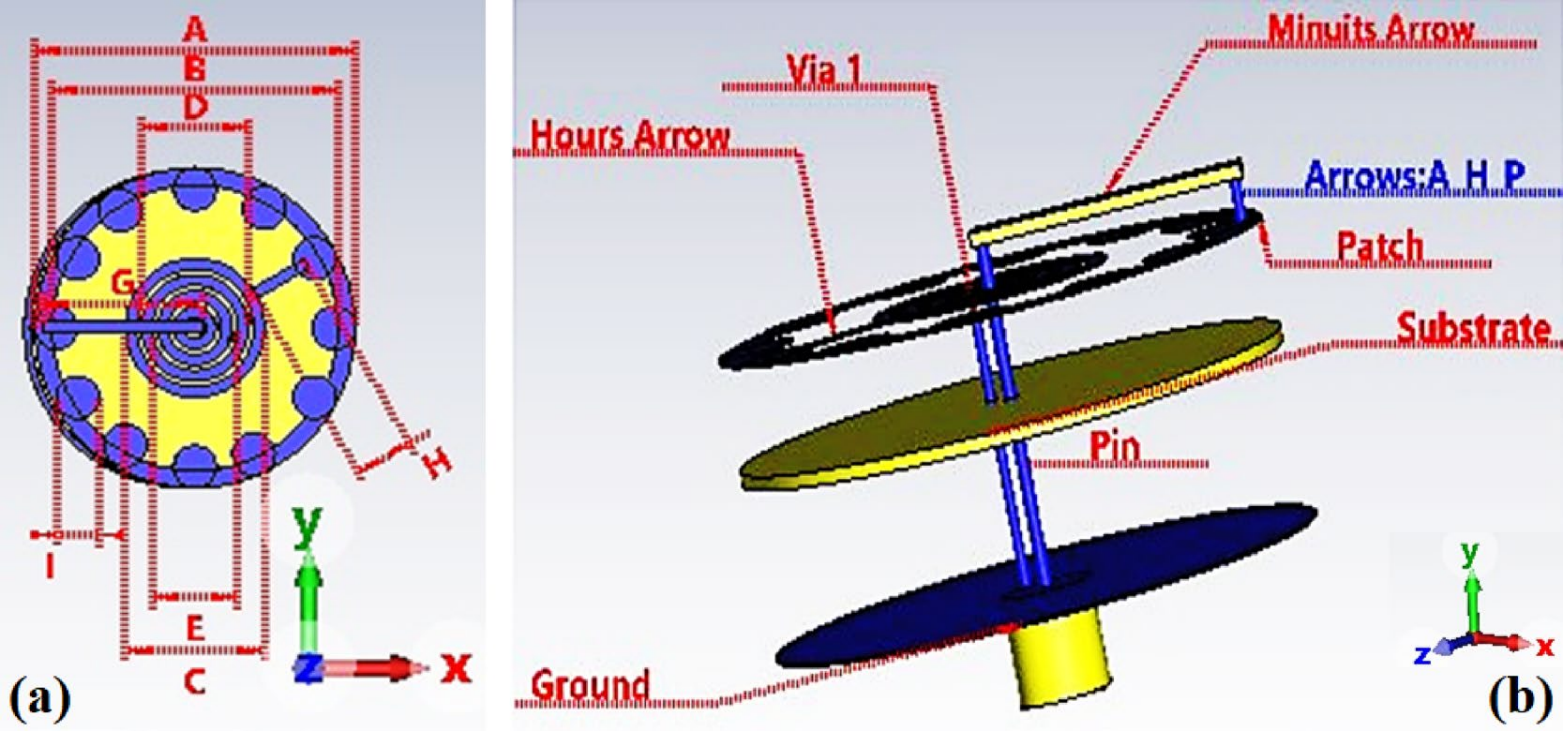
The proposed antenna structure, (**a**) Dimensions, (**b**) the feeder and short pin between the patch and ground.

Figure 1 illustrates the geometry of the watch antenna. All dimensions of the antenna are (diameter 30 × 0.635 height), and the antenna is composed of 2 copper-plated sheets. In this study, Roger’s RO6010 as substratum was utilized since its electrical characteristics ($\:{\epsilon\:}_{r}=10.5$ conductivity 0.0015 S/m, loss tangent is 0.0022). RO6010 was favored due to its easy availability and its compatibility with ordinary manufacturing procedures, making in-house prototype manufacturing possible.

The suggested tunable watch antenna is composed of a ground copper sheet. The substrate was sandwiched between the ground and the laminated copper sheet with a thickness of 0.005 mm. The hour’s arrow is etched inside the watch patch, while the minute’s arrow consists of three layers: ground, substrate, and patch, as illustrated in Fig. 1. The feeder connected to the minute’s arrow through the pin. The minute’s arrow connected to the watch patch through via2. The ground is connected to the watch patch via 1. So, the current path increases, which is responsible for the wide band. Changing the position of the minute’s arrow resulted in a new multiband antenna. If we manage to design a mechanism to control the watch arrows, then we have what is called a tunable antenna. Table 1 lists the dimensions of the proposed antenna. The hour and minute arrows, which are physically implemented as conductive structures within the patch, are rotated mechanically to provide tunability in the proposed watch antenna design. This modification alters the resonance frequencies and current pathways. Despite the absence of electronic tuning components such as varactors or MEMS, discrete tunability is provided by the manual positioning.

**Table 1:** Dimensions (mm) of the proposed Antenna.

A	B	C	D	E	F	G	H	I
30	27	13	10	8	13	15	6	4

The watch antenna, often referred to as a watch patch antenna, is a type of micro-strip antenna that includes a watch patch. The design and analysis of such antennas requires understanding the distribution of the electromagnetic fields, the resonant frequencies, and the impedance characteristics. However, the fundamental outline of the equations involved in the design of the proposed watch antenna can be given. To calculate the dimensions of the antenna, the following equations are used. For a simple micro-strip patch antenna, the resonant frequency f_0_ is given by:

1 $$\:{f}_{0}=\:\frac{1}{2\sqrt{{\epsilon\:}_{r}L}}\sqrt{\frac{c}{2L}}$$

where c is the speed of light in free space (3 × 10^8^ m/s), $\:{\epsilon\:}_{r}$ is the relative permittivity of the substrate, and L is the length of the patch.

The effective dielectric constant ($\:{\epsilon\:}_{r\:eff}$) can be approximated as:

2 $$\:{\epsilon\:}_{r\:eff}=\:\frac{{\epsilon\:}_{r}+1}{2}+\:\frac{{\epsilon\:}_{r}-1}{2}{\lceil\:1+12\frac{h}{W}\rceil\:}^{-\frac{1}{2}}$$

Where h is the height of the substrate, and W is the width of the patch. The dimensions of the patch (width W and length L) are given by:

3 $$\:w = \:\frac{c}{{2f_{0} }}\sqrt {\frac{2}{{\, \in \:_{r} + 1}}}$$

4 $$L = ~\frac{1}{{2f_{r} \sqrt { \in _{{reff}} } ~\sqrt {~\mu _{0} \varepsilon _{0} } }} - 2\Delta L$$

Where ΔL is the length extension due to fringing fields and is given by:

5 $$\:\frac{{\Delta \:L}}{h} = 0.412\frac{{\left( { \in _{{reff}} + 0.3} \right)(\frac{w}{h} + 0.264)}}{{\left( { \in _{{reff - 0.258}} } \right)(\frac{w}{h} + 0.8)}}$$

**Fig. 2:**
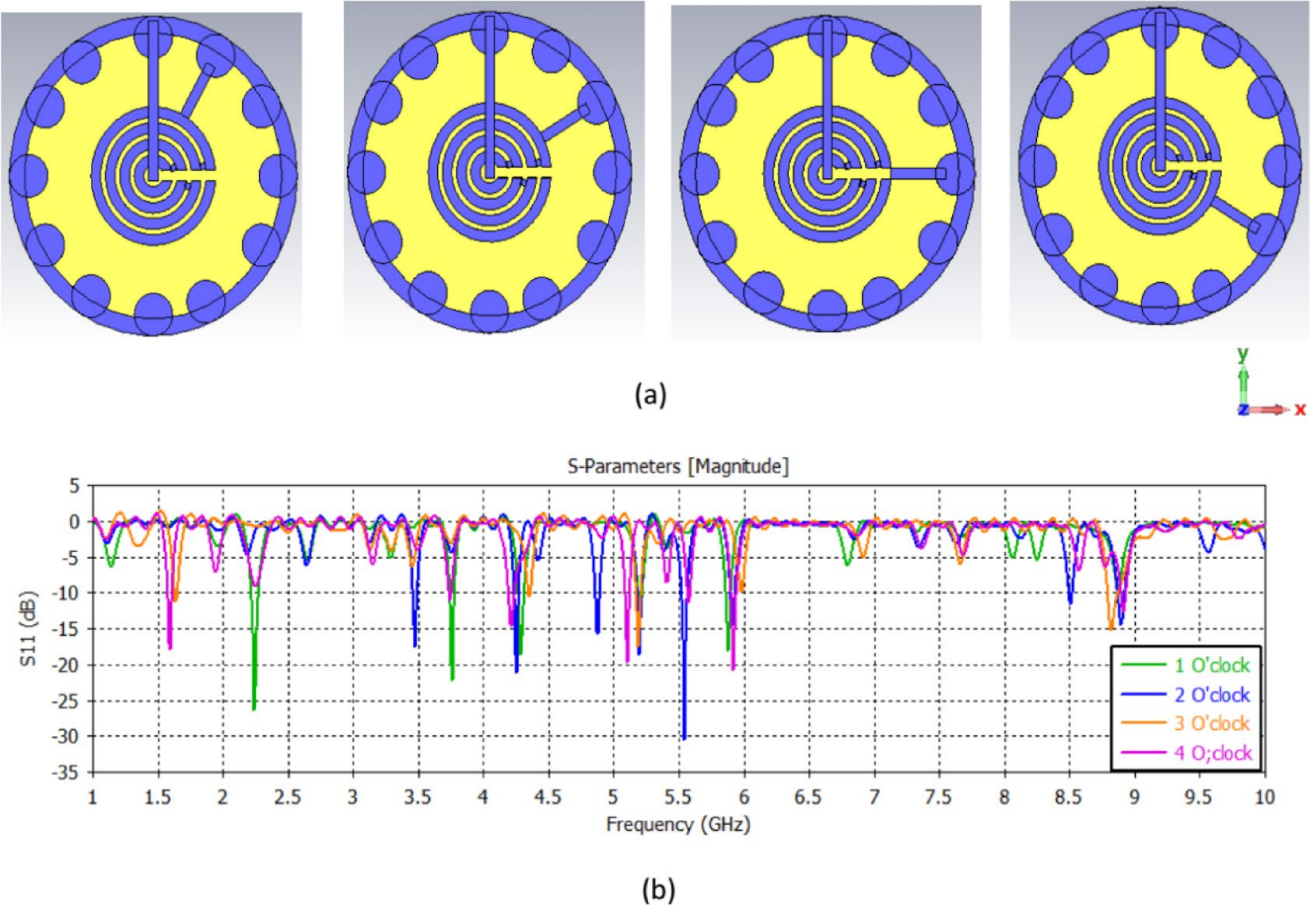
The simulation of the watch antenna in different positions of the minute’s arrow; (**a**) The structure, (**b**) The S_11_.

The watch antenna dimensions will vary according to the desired performance and tuning. The Circular, watch-shaped radius will affect the impedance bandwidth and can be optimized based on the obtained simulation results. In Fig. 2, the simulated scattering parameter S_11_ of the proposed antenna structure at various positions of the arrows, the antenna functions as a WBA with a broad operating frequency band from 0.1 to 10 GHz. Nonetheless, the antenna operates as a multi-band antenna. Although the proposed antenna has a wide operational frequency range (0.1–10 GHz), it functions as a reconfigurable multiband antenna in its manufactured configuration with the position of the arrows determining the band response.

When the arrow position is at the 1 o’clock position, the antenna resonates at frequencies (2.2 GHz, 3.7 GHz, 4.3 GHz, 5.2 GHz, 5.9 GHz) with S_11_ (-26 dB, -23 dB, -18 dB, 13dB, -17 dB) respectively. When the arrow position is at the 2 o’clock position, the antenna resonates at frequencies (1.6 GHz, 4.4 GHz, 5.1 GHz) with S_11_ (-11 dB, -10 dB, -17 dB) respectively. When the arrow position is at the 3 o’clock position; the antenna resonates at frequencies (3.5 GHz, 4.25 GHz, 4.9 GHz, 5.1 GHz, 5.5 GHz, 5.9 GHz) with S_11_(-17 dB, -21 dB, -16 dB, -18dB, -30 dB, − 15 dB) respectively. Finally, when the arrow position is at the 4 o’clock position, the antenna resonates at frequencies (1.55 GHz, 4.2 GHz, 5.1 GHz, 5.95 GHz) with S_11_ (-18dB, -16dB, -20dB, -21dB) respectively.

**Fig. 3:**
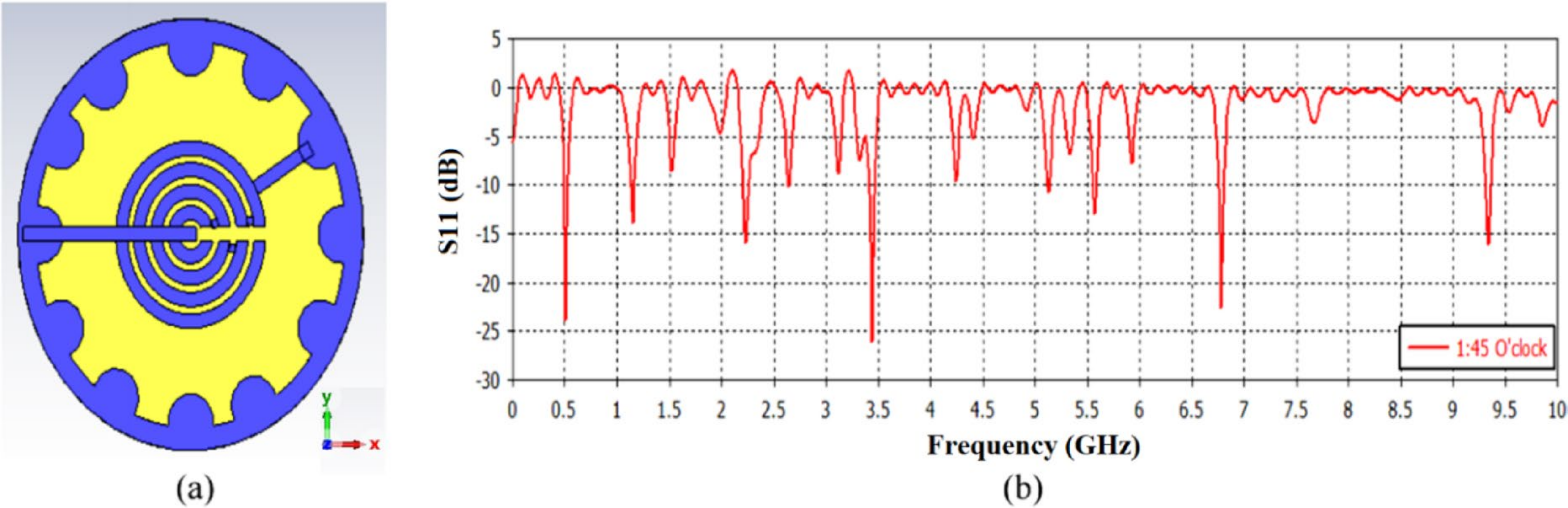
The proposed watch antenna at 1:45 O’clock (**a**) Structure, (**b**) S_11_.

The fabricated antenna was designed at 1:45 o’clock, and the resulting resonant frequencies were (0.5 GHz, 1.1 GHz, 2.1 GHz, 3.5 GHz, and 5.53 GHz) with S_11_ (-24 dB, -13 dB, -16 dB, -27 dB, -13 dB) respectively as shown in Fig. 3. Antenna manufacturing and the experimental measurements were performed at the National Telecommunication Institute (NTI), Cairo, Egypt. The raised noise in Fig. 3 is due to manufacture tolerances and connector reflections (e.g., inadequate linkages between the hours or minutes arrows and the main patch, resulting in interferences, reflections, and signal leakage), which were minimized during measurement calibration.

### Equivalent circuit of the proposed multiband watch antenna

An equivalent circuit analogy based on resonant LC circuits, which are frequently used to represent multiband and wideband antenna properties, can be created to demonstrate the multiband behavior of the proposed watch antenna. Lumped-element components (inductors, capacitors, and resistors) can be used to represent an antenna, with each resonant channel representing a distinct resonant frequency. Parallel RLC circuits, with each resonator tuned to a distinct frequency, are commonly used for multiband antennas. The hour and minute arrows on the watch antenna function similarly to changeable stubs or resonators. By generating varying electrical path lengths, their placements enable the antenna to be tuned to various frequencies. As a result, the equivalent circuit consists of several parallel RLC branches, each of which represents a distinct resonant frequency; a main feed line (represented by a transmission line or series RL component); and switchable stubs (minute/hour arrow positions), which simulate tunability based on arrow positions and are represented by varactor-tuned capacitors or variable inductors.

**Fig. 4:**
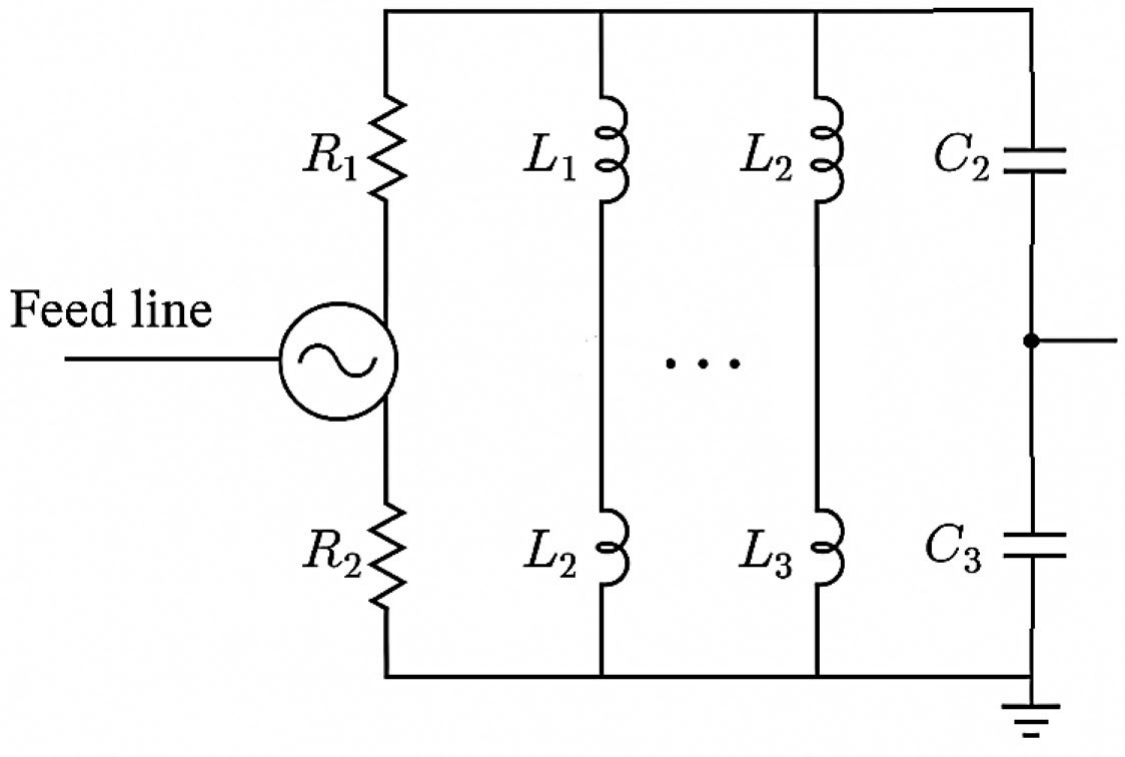
Equivalent circuit analogy for the antenna’s multiband behavior.

Equivalent circuit analogy for the antenna’s multiband behavior is presented in Fig. 4. Every branch (R, L, and C) exhibits resonance at a certain frequency range that is seen in both the simulation and the experiment. The minute/hour arrows’ changeable location can be represented as tuning or switching components (such as varactors or MEMS switches) that alter *L* or *C* to change the resonance. This parallel RLC resonator network simulates tunability through controllable L/C values (equivalent to changing arrow positions), allows multiple resonances, and provides physical insight into how changing geometrical configuration alters electromagnetic response, all of which successfully replicate the antenna’s observed multiband characteristics.

To model the resonance at the 1:45 configuration, we assumed a standard parallel RLC resonator model, where the resonant frequency is given by:

6 $$\:{f}_{0}=\frac{1}{2\pi\:\sqrt{LC}}$$

Assuming a standard capacitor value C = 1 pF, the corresponding inductance values for each resonant frequency will be as summarized in Table 2.

**Table 2:** Calculated Component Values for the Equivalent RLC Circuit of the Proposed Antenna.

Resonant Frequency (GHz)	Inductance LL (nH)	Capacitance CC (pF)	Resistance RR (Ω)
0.5	101.3	1	50
1.1	21.0	1	50
2.1	5.74	1	50
3.5	2.07	1	50
5.53	0.83	1	50

### Surface current distribution

**Fig. 5:**
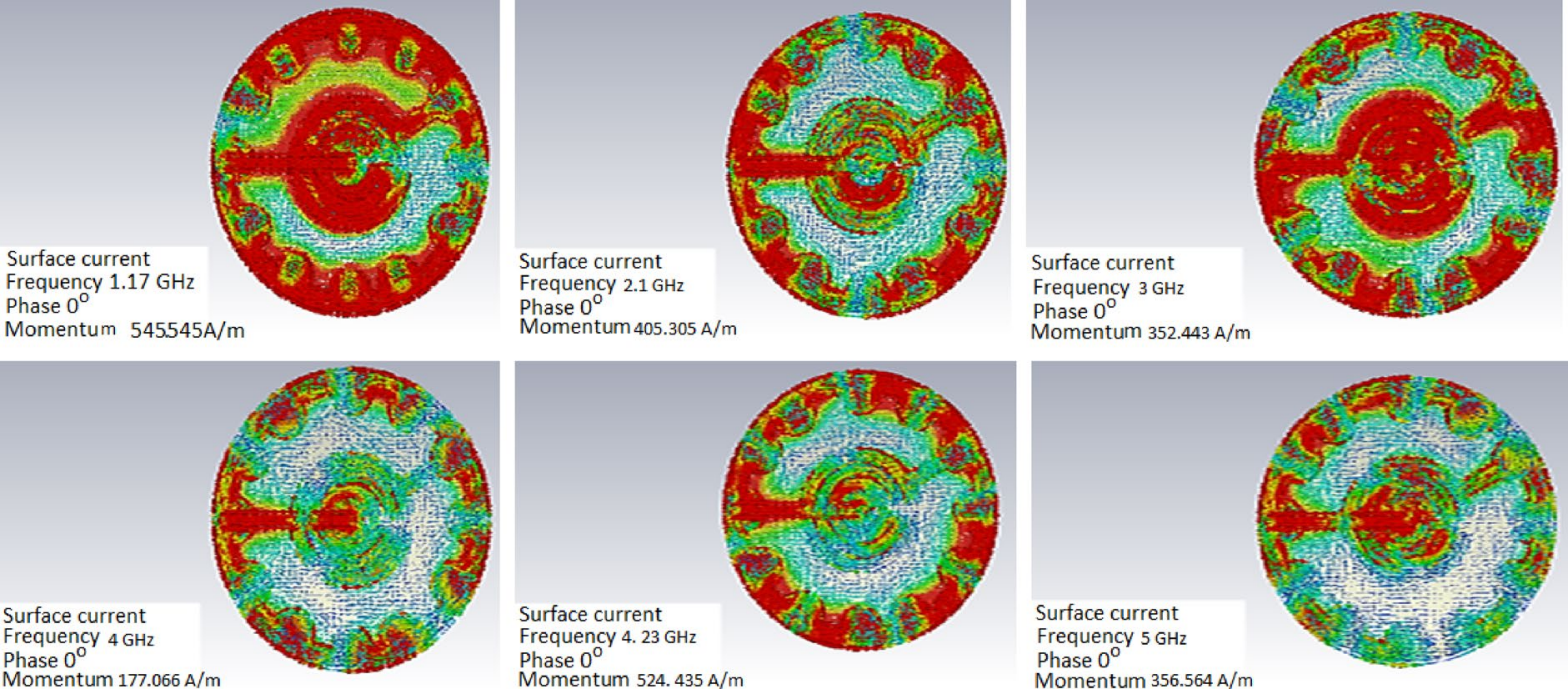
Current distributions for the 1:45 o’clock design at the operating frequencies.

Figure 5 shows the surface current distributions, which offer a better understanding of the design’s operation. Different resonating frequencies are obtained, ranging from 0.5 to 6 GHz, as shown by surface currents flowing along the edge of the feed patch and the center, watch-shaped structure. Different resonating frequencies were produced by the effect of the surface currents.

## Results and discussion

**Fig. 6:**
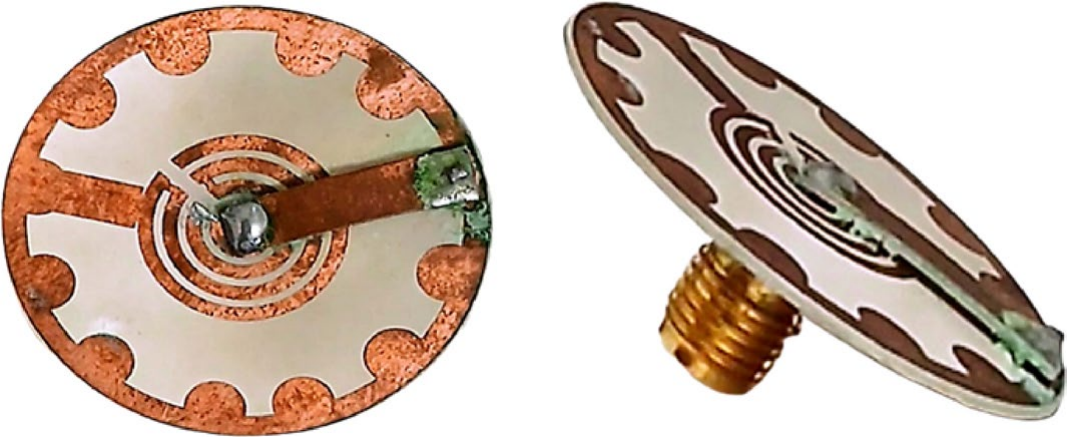
A real photograph of the fabricated antenna.

The experimental measurements of the antenna were implemented using a vector network analyzer (Rohde & Schwarz ZVB 20, 10 MHz—20 GHz). Figure 6 shows the prototype of the fabricated antenna, which was used in the measurement setup to characterize the manufactured prototypes and compute the radiation parameters in the azimuth and elevation planes.

### Reflection coefficient

**Fig. 7:**
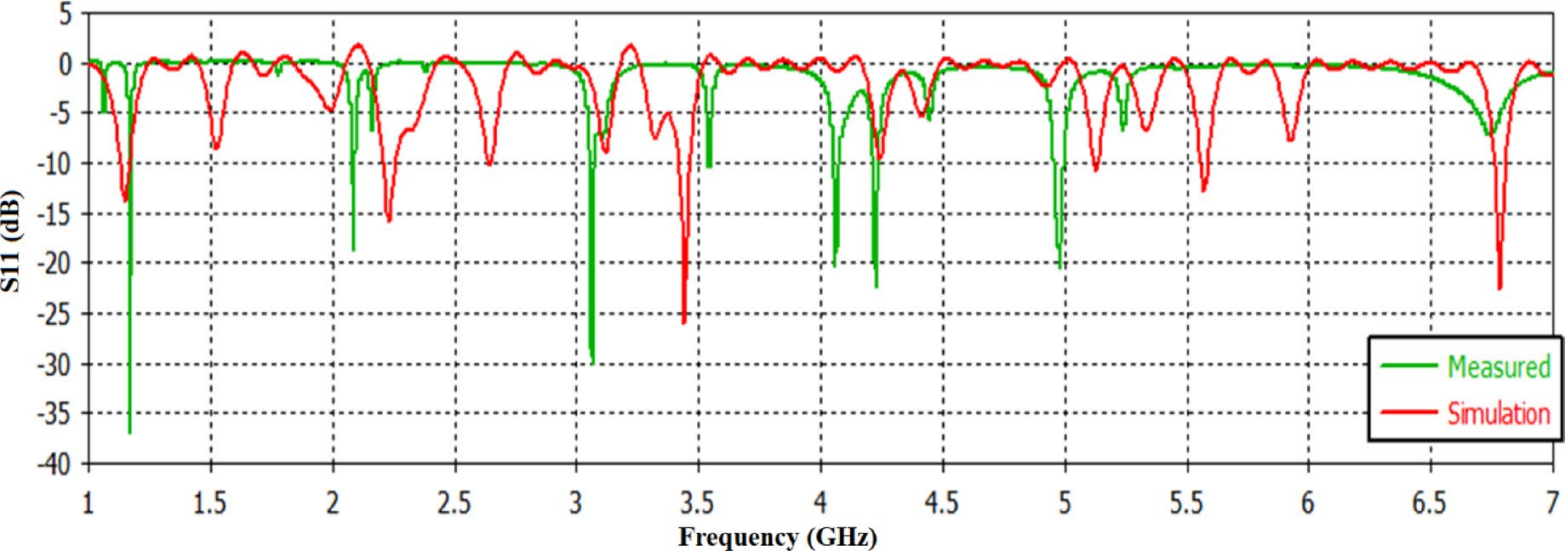
The S_11_ for the simulated and measured antenna.

Figure 7 presents the simulated and measured S_11_ for the antenna at 1:45 o’clock. The resulting simulated resonance frequencies were (1.1 GHz, 2.1 GHz, 3.5 GHz, and 5.53 GHz) with S_11_ ( -13dB, -16dB, -27dB, -13dB) respectively, while the measured resonance frequencies were (1.17, 2.09, 3.07, 3.52, 4.05, 4.2, 5) GHz respectively with corresponding S_11_(-37, -19, -29, -11, -20, -23, -20.5) dB respectively. The reduced bandwidth and deviation of the S11 in the measured response are mostly attributable to fabrication imperfections particularly the manual positioning of the watch arrows and the incorrect soldering procedure, which impacted impedance matching and current distribution. Additional issues like as material tolerances, higher loss, and measuring setup variability all contribute to the disparity. The apparent noise in the simulation is the result of incorrect connectivity between the hours or minutes arrows and the main patch, which caused interference. Table 3 summarizes the resonant frequencies and corresponding S11 values calculated using CST simulation, physical measurements, and the equivalent circuit model The equivalent circuit model properly duplicates the resonant behavior observed in both simulation and measurement, confirming the parallel RLC analogy’s validity for multiband performance. There was a shift in the operating frequencies in the fabricated antenna to the left; this is due to the fixation of the arrows and the errors resulting from the soldering process.

**Table 3:** Summary of Simulated, Measured, and equivalent circuit S11 parameters for the watch antenna at 1:45 o’clock Configuration.

Frequency band		Frequency (GHz)	S_11_ (dB)	Band width (MHz)
1	Simulated	1.15	– 14	105
	Measured	1.17	– 37	25
	Equivalent Circuit	1.15	– 12.5	–
2	Simulated	2.23	– 16	98
	Measured	2.09	– 19	20
	Equivalent Circuit	2.09	– 15.2	–
3	Simulated	3.12	– 10	30
	Measured	3.07	– 29	39
	Equivalent Circuit	3.10	– 13.1	–
4	Simulated	3.48	– 26	65
	Measured	3.52	– 11	15
	Equivalent Circuit	3.50	– 23.5	–
5	Simulated	4.2	– 10	20
	Measured	4.05	– 20	15
	Equivalent Circuit	4.10	– 14.8	–
6	Simulated	4.45	– 5	22
	Measured	4.2	– 23	17
	Equivalent Circuit	4.30	– 11.5	–
7	Simulated	5.13	– 11	25
	Measured	5	– 20.5	20
	Equivalent Circuit	5.05	– 10.7	–

### Radiation patterns

**Fig. 8:**
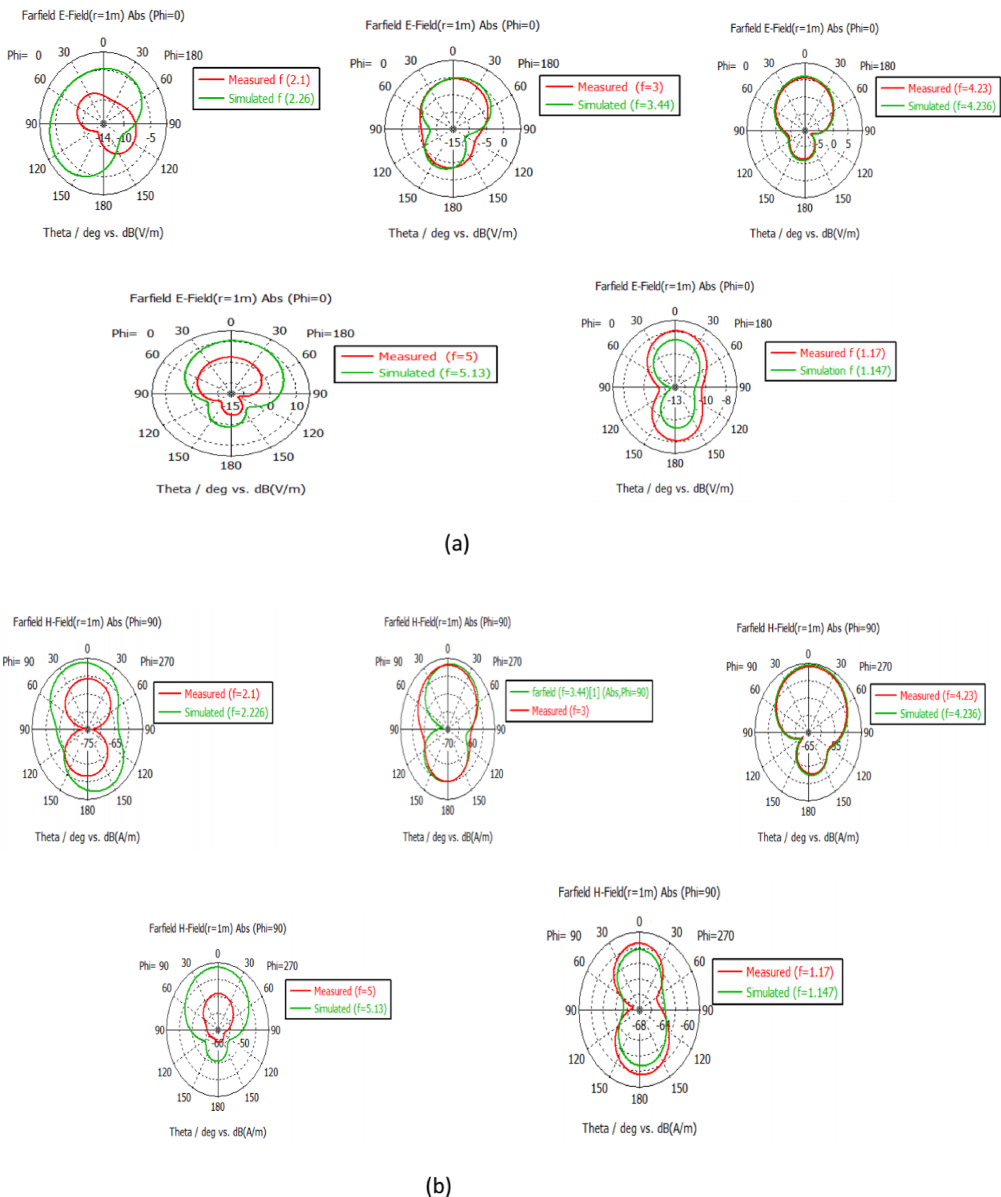
The radiation pattern for the simulated and measured antenna. (**a**) The E-plane radiation pattern (**b**) The H-plane radiation pattern.

Figure 8 shows the simulated and measured 2D radiation patterns for the proposed antenna at resonant frequencies. Figure 8 shows the computed 3D radiation patterns for the proposed antenna at 1:45 o’clock. The radiation patterns in the H- and E-planes show plots of cross- and co-polarization, respectively. The excellent coincidence between the generated and measured patterns is evident. The discrepancies between the simulated and measured findings could be due to the errors resulting from the connectors’ soldering and manufacturing processes.

**Fig. 9:**
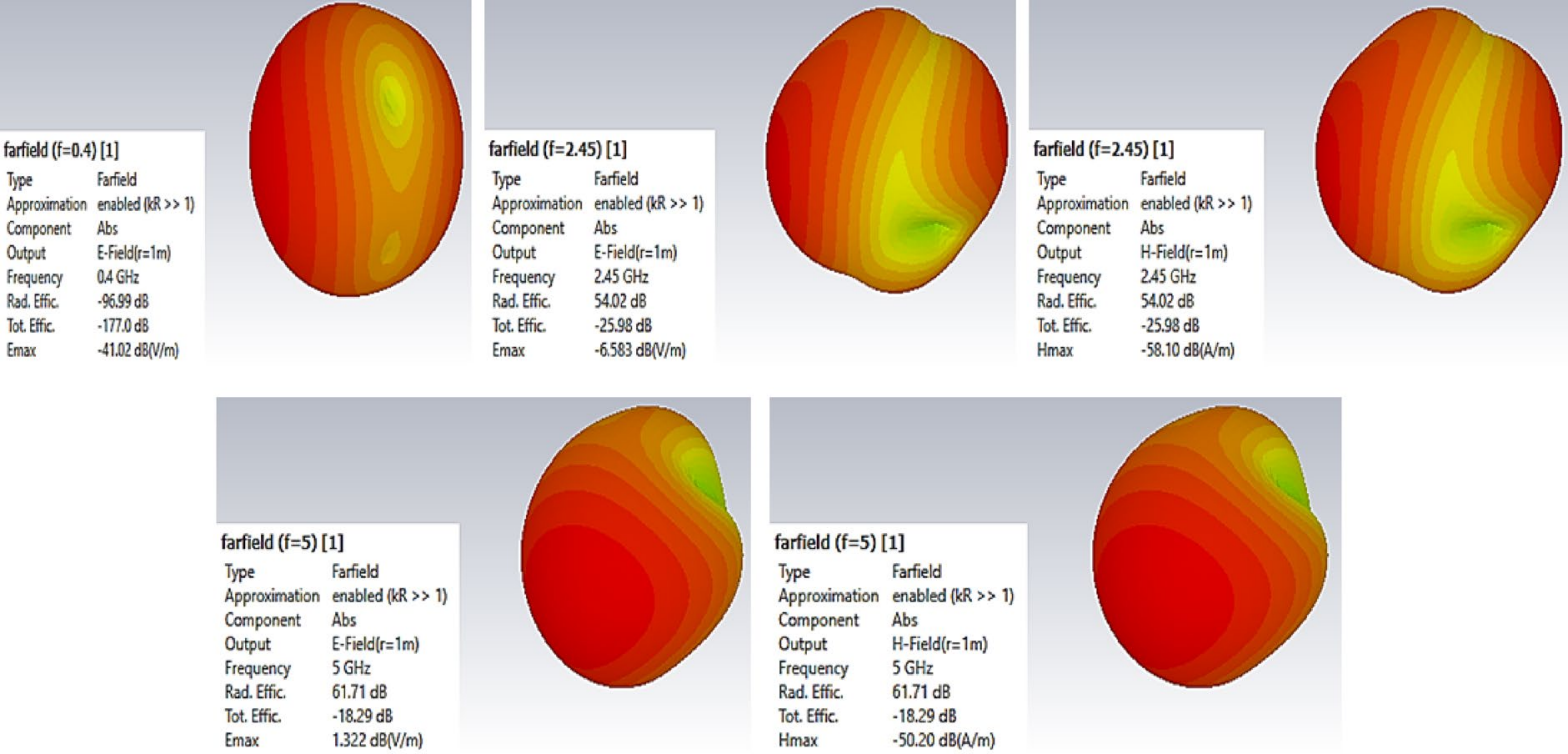
The 3D radiation pattern for the proposed antenna.

The 3D-radiation pattern images for the resonant frequencies are presented in Fig. 9. The distribution and concentration of radiated power in various directions clearly show these patterns. The graphs show the radiation properties of the antenna at the resonant frequencies in a visual manner.

### Gain, efficiency, and axial ratio

**Fig. 10:**
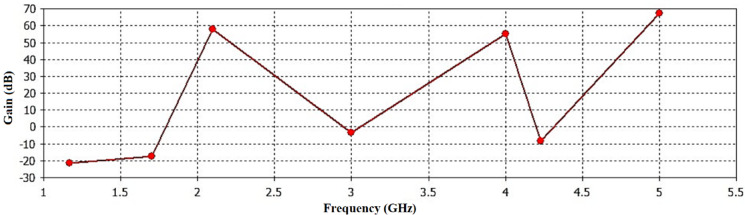
The reported gain of the proposed antenna.

Figure 10 displays the proposed antenna’s attained gain. The maximum obtained gain was 70 dB at frequency 5 GHz, while at 2.22 GHz the gain was 60 dB. At 4 GHz the obtained gain was 55 dB.

**Fig. 11:**
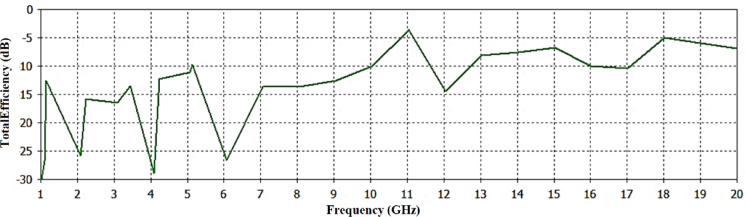
Efficiency of the proposed antenna.

Furthermore, Fig. 11 shows that the total efficiency of the proposed antenna was calculated. The antenna shows maximum efficiency of − 3.8 dB or 42%.

Axial Ratio (AR) of an antenna is defined as the ratio between the major and minor axes of a circularly polarized antenna pattern.

$$\:AR\left(dB\right)=20\:.\:{log}_{10}\:\left(\frac{Major\:Axis}{Minor\:Axis}\right)$$

**Fig. 12:**
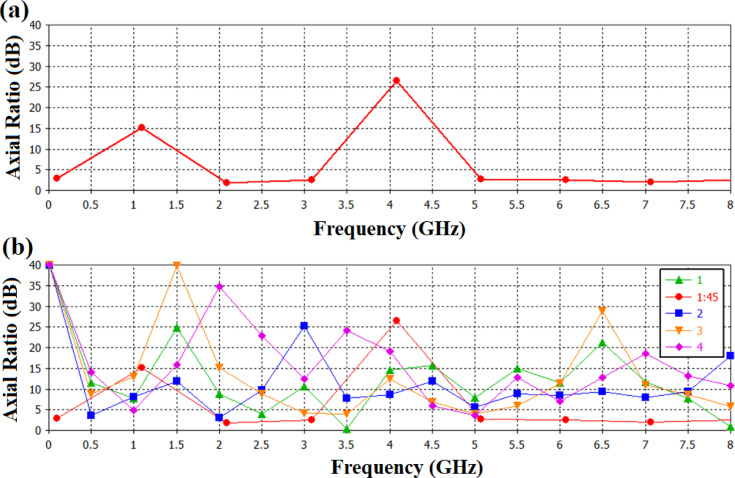
Axial Ratio for the proposed antenna (**a**) Measured antenna, (**b**) Covering all the bands of interest.

If an antenna has perfect circular polarization, then this ratio would be 1 (0 dB). In addition, the axial ratio tends to degrade away from the main beam of an antenna. The axial ratio (AR) has been investigated in all operating bands in order to confirm the circular polarization properties of the proposed antenna. With AR values below the 3 dB threshold (a crucial need for circularly polarized antennas) the antenna exhibits satisfactory circular polarization performance. As shown in Fig. 12.a. The AR remains between 2.7 and 2.9 dB in the lower band (2.1–3.15 GHz), showing constant circular polarization within ± 30° of the primary beam direction. The whole AR spectrum is shown in Fig. 12(b), confirming that all resonance frequencies of relevance satisfy the circular polarization specifications needed for realistic wireless applications.

**Fig. 13:**
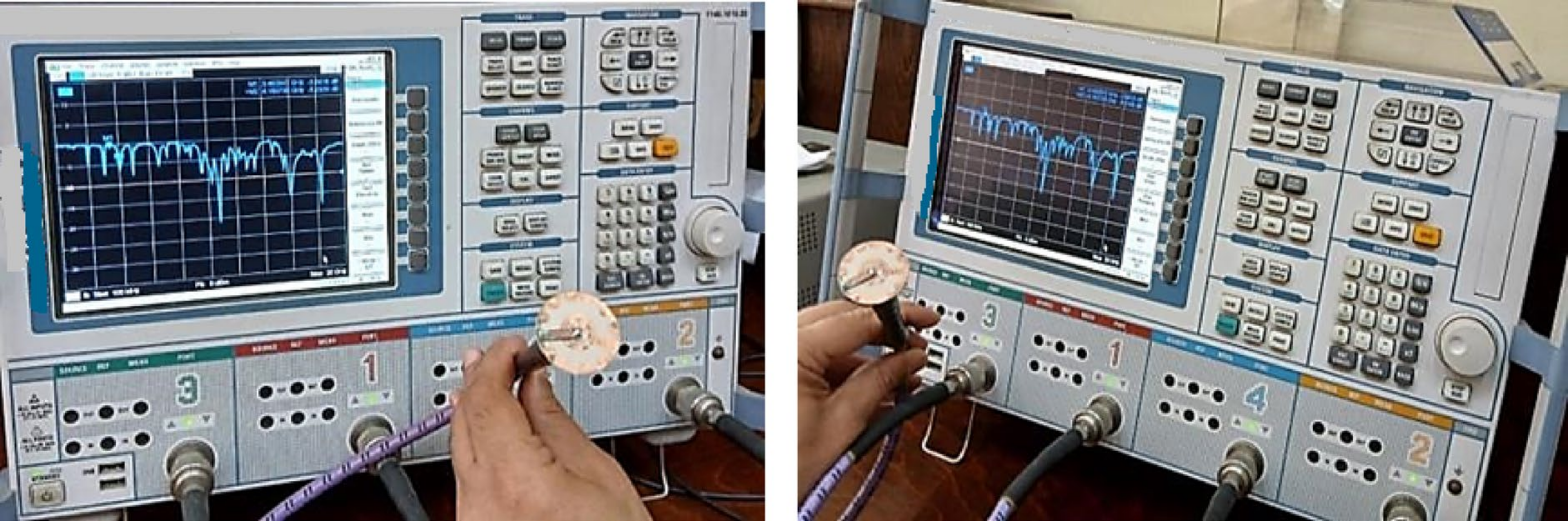
Measurement setup of the proposed antennas using VNA.

Figure 13 shows the measuring apparatus, the Vector Network Analyzer (VNA).

## Comparison with related work

Despite attempts to produce a multiband antenna with outstanding performance, design considerations like the low profile and improved gain make it challenging to sacrifice the compact size. In this paper, a multilayer multiband watch antenna is introduced. As the most important factors for wireless communications applications, different parameters were studied when developing the proposed structure for GSM, CDMA, UMTS, LTE, Wi-MAX, and 5G (Ultra-Fast & Low Latency) communications applications. In comparison to the works presented in the papers from 1 to 6 listed in Table 4, the proposed multi-band watch antenna exhibits excellent features and performance in terms of reflection coefficient, the operating bandwidth (GHz), and gain (dB).

**Table 4:** Comparison with related works.

No.	Ref	Size	Operating band (GHz)	Gain (dBi)	Efficiency (%)
1	^19^	0.46 λ0 × 0.29 λ0	2.3-3 3.25–3.68 4.9–6.2	4.32	–
2	^26^	0.24 λ0 × 0.18 λ0	3.1–10.6	2.5	–
3	^27^	0.38 λ0 × 0.34 λ0	2.34–2.82 3.16–4.06 4.69–5.37	3.02	–
4	^32^	0.9 λ0 × 0.78 λ0	4.27-5.5- 7.2	2.1	80- 97- 40
5	^35^	0.39 λ0 × 0.28 λ0	3.5–3.75 4.85–5.2 5.5–5.7	8	–
6	^36^	0.35 λ0 × 0.35 λ0	1.8–3.7 4.05–5.5	8.5	90
7	This work	0.035 λ0 × 0.0007 λ0	1.17-2.09- 3.07- 3.52- 4.05- 4.2- 5	6	42 (-3.8 dB)

As summarized in Table 4, the proposed antenna features a mechanically tunable multiband design with a compact footprint of 30 mm diameter and 0.635 mm thickness. compared to traditional reconfigurable antennas, which rely on electrical components, the antenna uses manual rotation of watch-inspired hour and minute arrows to change current routes and resonant frequencies. This design supports separate tuning across seven frequency bands ranging from 1.17 to 5 GHz. The antenna has a peak gain of up to 70 dB and a maximum measured efficiency of 42% (-3.8 dB), making it appropriate for modern wireless applications such as GSM, CDMA, UMTS, LTE, Wi-MAX, and sub-6 GHz 5G. Its low profile, ease of manufacture, and reconfigurability make it a good option for compact wearable or embedded wireless systems.

## Conclusions

In this study, we present the design, simulation, development, and testing of an innovative watch-shaped multiband antenna that can be adjusted to suit contemporary wireless applications. Dynamic control over resonant frequencies is made possible by the new structure’s mechanically adjustable hour and minute arrow elements. The antenna’s ability to handle numerous bands in the 1–6 GHz range, including important wireless technologies like GSM, CDMA, UMTS, LTE, Wi-MAX, and sub-6 GHz 5G, is confirmed by simulation and manufacturing findings.

## Data Availability

The datasets analyzed during the current study are available from the corresponding author on reasonable request.
